# Insights on Development Aspects of Polymeric Nanocarriers: The Translation from Bench to Clinic

**DOI:** 10.3390/polym14173545

**Published:** 2022-08-29

**Authors:** Akhilesh Kumar Tewari, Satish Chandra Upadhyay, Manish Kumar, Kamla Pathak, Deepak Kaushik, Ravinder Verma, Shailendra Bhatt, Ehab El Sayed Massoud, Md. Habibur Rahman, Simona Cavalu

**Affiliations:** 1 M.M. College of Pharmacy, Maharishi Markandeshwar (Deemed to be University), Mullana, Ambala 133207, Haryana, India; 2 Formulation Research and Development, Mankind Research Centre, Manesar, Gurugram 122050, Haryana, India; 3 Faculty of Pharmacy, Uttar Pradesh University of Medical Sciences, Saifai, Etawah 206130, Uttar Pradesh, India; 4 Department of Pharmaceutical Sciences, Maharshi Dayanand University, Rohtak 124001, Haryana, India; 5 Department of Pharmacy, G.D. Goenka University, Sohna Road, Gurugram 122103, Haryana, India; 6 Biology Department, Faculty of Science and Arts in Dahran Aljnoub, King Khalid University, Abha 62529, Saudi Arabia; 7 Research Center for Advanced Materials Science (RCAMS), King Khalid University, Abha 61413, Saudi Arabia; 8 Agriculture Research Centre, Soil, Water and Environment Research Institute, Giza 3725004, Egypt; 9 Department of Global Medical Science, Wonju College of Medicine, Yonsei University, Wonju 26426, Gangwon-do, Korea; 10 Faculty of Medicine and Pharmacy, University of Oradea, P-ta 1 Decembrie 10, 410087 Oradea, Romania

**Keywords:** polymeric nanocarriers, polymersomes, nanosized hydrogels, cubosomes, ocular, parenteral

## Abstract

Scientists are focusing immense attention on polymeric nanocarriers as a prominent delivery vehicle for several biomedical applications including diagnosis of diseases, delivery of therapeutic agents, peptides, proteins, genes, siRNA, and vaccines due to their exciting physicochemical characteristics which circumvent degradation of unstable drugs, reduce toxic side effects through controlled release, and improve bioavailability. Polymers-based nanocarriers offer numerous benefits for in vivo drug delivery such as biocompatibility, biodegradability, non-immunogenicity, active drug targeting via surface modification, and controlled release due to their pH—and thermosensitive characteristics. Despite their potential for medicinal use, regulatory approval has been achieved for just a few. In this review, we discuss the historical development of polymers starting from their initial design to their evolution as nanocarriers for therapeutic delivery of drugs, peptides, and genes. The review article also expresses the applications of polymeric nanocarriers in the pharmaceutical and medical industry with a special emphasis on oral, ocular, parenteral, and topical application of drugs, peptides, and genes over the last two decades. The review further examines the practical, regulatory, and clinical considerations of the polymeric nanocarriers, their safety issues, and directinos for future research.

## 1. Introduction

A polymer represents several repeating units leading to the formation of a compound that has characteristics of high relative molecular mass and associated properties. In 1833, Jons Jakob Berzelius coined the term “polymer” which originated from the Greek word (polus, meaning “many, much”) and (meros, meaning “part”). Following major developments in polymer fabrication, the molecular properties of polymers were only recognized after the efforts of Hermann Staudinger in 1922, who first suggested that polymers consist of long chains of atoms bound together by covalent bonds. This took more than a decade to achieve acceptance among the scientific community. Initially, polymers were investigated according to the theory of associations or composite theory that emerged in 1861. It was suggested that cellulose and other polymers were colloids and molecular aggregates with small molecular weights bound by an unidentified intermolecular force. The use of polymers is not new in the medical field [1].

Natural polymers are being used for decades as ingredients for herbal remedies. The case is very different when it comes to synthetic polymers. Water-soluble polymers can be considered a modern accomplishment as macromolecular drugs or as part of the inoculation-related drug delivery systems. The first polymer-drug conjugate in form of mescaline-N-vinylpyrrolidone conjugates was synthesized in 1955. A decade later, it was predicted that polyethylene glycol (PEG) can be attached to proteins using a method called PEGylation that comprises adding the covalent bond of polymer (ethylene glycol) polymer chains to some other entity, normally a therapeutically active drug or protein. In 1994, the first synthetic polymer-drug conjugate was clinically proven to treat cancer. This was made up of a doxorubicin copolymer conjugation of (N-(2-hydroxypropyl) methacrylamide [2]. In 1977, Davis and Abuchowski described first-time PEGylation for drug delivery via the covalent attachment of PEG to bovine serum albumin and liver catalase proteins [3]. They reported that it could increase the systemic circulation time and decrease the immunogenicity of the proteins without significantly compromising activity. In 1990, the FDA approved the first PEGylated protein product, Adagen^®^, a PEGylated adenosine deaminase enzyme for severe combined immunodeficiency disease. Doxil^®^ is the first FDA-approved PEGylated nanoparticle (NP) based product that was commercialized in 1995. At present, eight other PEGylated protein therapeutics have been FDA approved for various diseases [4]. The success of protein PEGylation as a method for producing longer circulating, and thus more efficacious intravenous therapies, led to investigations of nanoparticle (NP) PEGylation for systemic applications in the early 1980s and 1990s [5]. Recognized as foreign objects, they are readily excreted from the systemic circulation by the cells of the mononuclear phagocyte system, precluding accumulation in target cells and tissues [6,7,8]. Block copolymer-based micelles have also been explored for targeting specific cancer cells as these micelles are capable of trapping the drug or connecting it covalently to the co-polymers. Two polymer-protein complexes, PEG-interferon-α (an antiviral medication meant to treat liver cirrhosis) and PEG granulocyte colony-stimulating factor were put on the market around the 2000s [9]. The first therapeutic nanoparticles (albumin-trapped paclitaxel) were licensed for metastatic breast cancer treatment after five years [10]. Since then, polymeric micelles and nanoparticles comprising block copolymers have emerged as a storm and impacted clinical care. Some of the polymeric micelles have been approved and are currently being marketed in Korea and Europe. Genexol-PM is a polymeric micellar formulation of paclitaxel got approved in 2007 and is marketed under the name Cynviloq by Sorrento Pharmaceutics [11]. In 2013, NanoCarrier Co. Ltd. Tokyocompleted Phase II clinical studies of Nanoplatin (NC-6004) which is a polymeric micellar formulation of cisplatin [12]. NK012 of Nippon Kayaku Co. Ltd. Tokyo, is another polymeric micelle formulation of SN-38 (an irinotecan metabolite) which has completed Phase II clinical trials in 2015 and received orphan drug designation from USFDA [13]. In 2017, Nippon Kayaku Co., Ltd., Tokyo completed another clinical Phase III study for NK105 which is a polymeric micellar formulation of paclitaxel consisting of PEG and modified polyaspartate as a hydrophobic block. Currently, Docetaxel-loaded polymeric micelles plus Oxaliplatin is under Phase II clinical trial for patients with esophageal squamous cell carcinoma. Epirubicin-loaded polymeric nanoparticles is undergoing a Phase I/II clinical trial for patients having tumors or soft tissue sarcoma. Another polymeric nanoparticle loaded with Cetuximab is undergoing a Phase I clinical trial for colon cancer [14]. All of the above accomplishments represent the central factor that led to the growth of polymer-based pharmaceutical products, viz. nanoparticles, nanoconjugates, and micelles.

Nanomaterials took the pharmaceutical, nutritional supplements, and food industries by storm. Such structures are 1–100 nm (10^−9^ m) in diameter and have distinct physicochemical properties in comparison to macromaterials [15]. Nanotechnology is one of the most innovative technologies and is capable of improving food safety and quality. However, there are many studies showings that nanomaterials are not inherently harmless [16]. Nanomaterials are used for therapeutics in the healthcare sector, treatment for cancer, and diagnosis of disease. But in addition to their advantages, they also possess several undesirable effects, among which an increase in the absorption rate is the most important [17,18,19]. They increase the absorption rate as a result of the increased surface area of the nanomaterials, which allows increased absorption rate by the surrounding tissues leading to the generation of several unintended results [20]. The increased absorption rate is often seen in powder nanomaterials but less so in solids. However, nanomaterials are used to minimize these effects through controlled absorption [21]. Polymers possess unique qualities, including biocompatibility and biomimetic properties, which is why they are widely applicable in biomaterials as biosensors, catalysis, and the delivery of medicines [22]. Their use in medicines is beneficial due to their ability to distribute the therapeutic compound efficiently to the active site or target, making drug safety and efficacy certain [23].

## 2. Types of Polymer Based Nanocarriers

**Figure 1:**
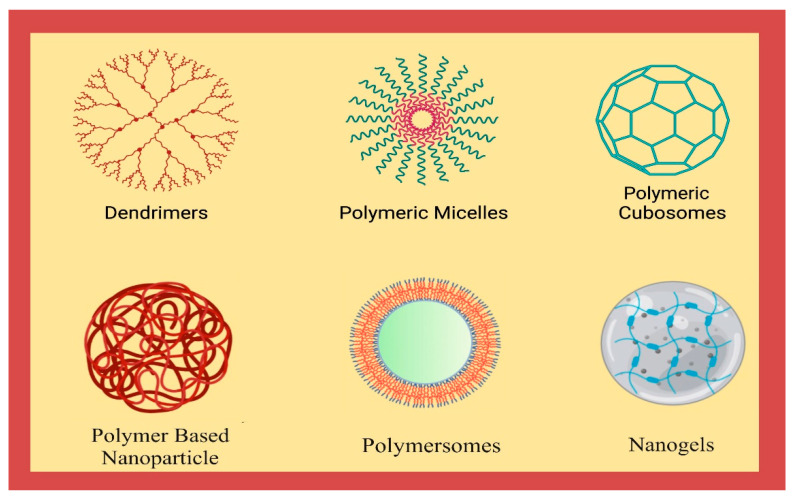
Diagrammatic representation of different polymeric nanocarriers.

The precise classification of the nanocarriers is a complicated task to attempt. In the arena of biomedical and pharmaceutical nanotechnology, no specific boundaries have been defined, and therefore different perceptions may be relevant. However, a concise sketch of potential polymer-based nanocarriers is important as depicted in Figure 1. Depending on their origin they can also be identified as natural and synthetic polymeric nanoparticles. These are distinguished by their concurrent polymeric design and colloidal (1–1000 nm) measurements and have been commonly identified as polymeric (biodegradable and biocompatible) nanoparticles, dendrimers, nanoemulsions, polymersomes, polymeric micelles, or biopolymer complexes or cubosomes [24].

The highly relevant articles were retrieved via various search engines on the databases, Science Direct, Web of Sciences, PubMed, Scopus, PubChem, and Google Scholar. The examples have been selected on the basis of their significance to provide experimental insight into each type of nanocarriers to the readers. The keywords and phrases used for the search are “dendrimers”, “polymeric micelles”, “polymerosomes”, “nanogels”, “dendrimers”, “cubesomes”, “NLCs”, “nanographene”, “nanocomposites”, “CNTs”, “polymers”, and “nanocarriers”, “In vitro and in vivo activities”. The inclusion criteria implied for selecting particular polymer-based nanocarriers represent the significance of that particular study.

### 2.1. Polymer-Based Nanoparticles

Nanoparticles are sub-nanosized colloidal structures composed of synthetic or semisynthetic polymers. NPs range in size between 1 and 100 nm [25]. The ultra-small size of NPs exhibits a larger surface area to volume ratio and hence allows greater drug loading within the core via encapsulation and onto the surface via absorption. Their unique size range allows for extended circulation, leading to prolonged drug release. The entrapment of drugs in NPs protects against chemical degradation and improves their pharmacokinetic properties, leading to controlled release over a long period. NPs exhibits flexibility of surface functionalization with targeting ligands for site-specific drug delivery and thereby improve therapeutic efficacy [26]. Materials used in the manufacturing of nanocarriers based on polymers are selected not only for their functional characteristics, such as their capability to structure planned nanostructures, but also for their biological behavior. Polymers that are biodegradable and biocompatible are usually favored. Given the multitude of accessible or easily synthesizable polymers with promising potential for medicinal use, regulatory approval has been achieved for just a few. Various categories and forms of polymers including poly-lactic-co-glycolic acid (PLGA), poly-DL-lactic acid (PDLLA), polycaprolactone (PCL), polyacrylate, polymethacrylate, cellulose by-products, poly(ethylene oxide) triblock (PEO)/polypropylene oxide (PPO) (PEO-PPO-PEO; poloxamers), PEG, polyvinyl alcohol (PVA) and alginate was licensed by the United States Food and Drug Administration (USFDA) and other foreign authorities for various medical purposes over the years [27,28].

However, not all of these have sufficient features to develop nanomaterials that can act as nanocarriers, so unsurprisingly previously authorized materials may be standard candidates for rapid clearance by regulatory agencies. In comparison, modern polymer conjugates and polymers need rigorous biological and toxicological assessment to be approved for human use, and their clearance may be overwhelming [29].

Yaşar et al., 2020 developed polymer-based NPs with 74 nm particle size for selective protein recognition by using thiol-ene miniemulsion photopolymerization. The developed NPs revealed more selectivity toward myoglobin [30].

Puri et al., 2017 fabricated pH-sensitive polymer-based NPs to deliver bioactive compounds employing a dispersion polymerization approach. They reported that the rate of hydrolysis and drug discharge were quicker at pH 5.0 compared to pH 7.4 [31].

### 2.2. Polymeric Micelles

Fabricated by amphiphilic block copolymers, the polymeric micelles can be self-assembled (and disassembled) under a valid concentration or temperature environment as versatile spherical structures (i.e., nanosized core-shell molecules produced by self-association of amphiphilic block copolymers when exposed to an aqueous solvent). In nature, the core of micelles is hydrophobic and appropriate for incorporating hydrophobic moieties [32]. They are suitable for parenteral delivery compared to current solubilizing agents (e.g., cremophor EL), causing the dosage of effective but toxic and poorly water-soluble compounds to increase [33]. Investigations into nano-bio dynamics which can function at the molecular, cellular, and tissue levels is often ignored [34].

**Figure 2:**
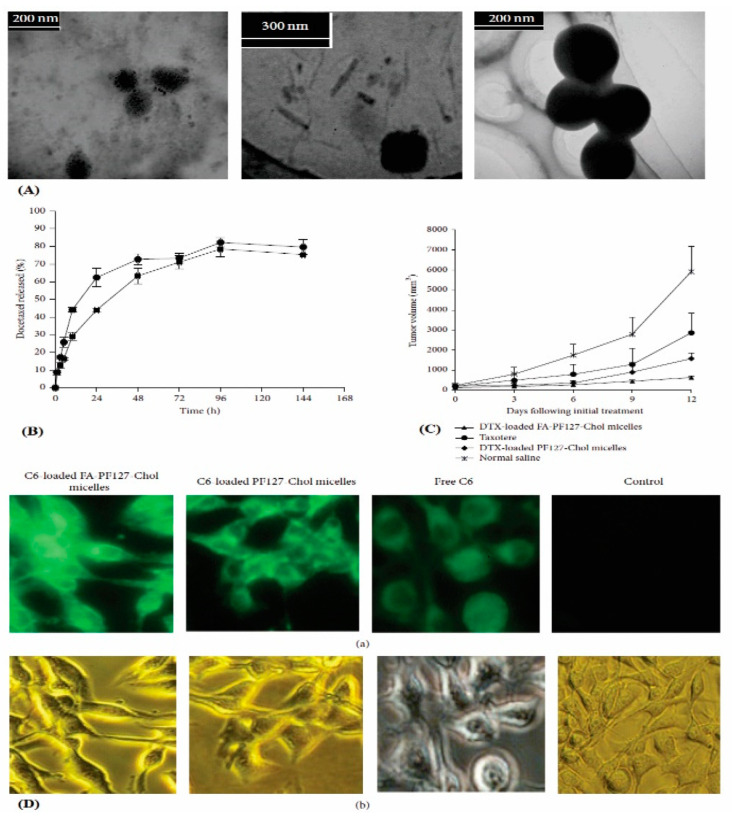
Illustration of (**A**) TEM analysis; (**B**) in vitro drug release studies; (**C**) in vivo antitumor efficacy studies on B16F10 melanoma bearing mice; (**D**) Results of in vitro cellular uptake studies of C6 loaded FA-targeted PF127 Chol micelles, C6 loaded non-targeted PF127 Chol micelles, free C6, and culture media (Control) via (a) fluorescent microscopy and (b) visible light microscopy. Reproduced from Varshosaz et al., 2015 [35] under creative commons CC BY license *©* 2015, The Author(s).

Varshosaz et al., synthesized docetaxel-loaded polymeric micelles using folic acid grafted synperonic PE/F 127-cholesteryl hemisuccinate copolymer for precise delivery to cancerous cells. The in vitro cellular uptake observations in B16F10 melanoma cells revealed improved uptake of targeted polymeric micelles through folic acid receptor-mediated endocytosis [35]. The micelles were found to be non-toxic and safe for biomedical usage over a range of 0.001 to 500 µg/mL. The morphological examination revealed the smooth spherical shape of the micelles. In vitro drug release investigation showed an initial burst trailed by slower and sustained release up to 134 h. The early burst release could be credited to the quick dissolution of surface-bound drugs followed by steady diffusion from the core of micelles. An in vivo antitumor efficiency study in B16F10 melanoma-bearing mice revealed effectively inhibited tumor growth by the drug-loaded targeted polymeric micelles compared to non-targeted polymeric micelles as shown in Figure 2.

Chida et al., fabricated epirubicin (EPI) loaded polymeric micelles armed with pH-sensitive drug release characteristics in contradiction to axillary lymph node metastasis (ALNM) of triple-negative breast cancer (TNBC) for improving the therapeutic effect. By accumulating selectively and penetrating both the vascularized ALNM and primary tumor, as well as effective drug trigger caused by intratumoral acidic atmosphere, the proposed formulation successfully prevented the growth of ALNM and the expansion of the primary tumor [36].

Bae et al., designed environment-sensitive supramolecular structures for intracellular drug delivery of Adriamycin (ADR) via linking it to the polymer by a pH-sensitive coupler. The micelles treated carcinoma cells via their transportation into a cell by endocytosis, consequently avoiding cell-membrane transporters [37].

### 2.3. Polymersomes

Polymersomes are considered the next era of nanomedicine for clinical diagnosis and therapies due to their potential for controlling structure and features. These are the most favorable tools for a biomedical utility such as diagnosis and controlled drug delivery due to the compartmentalization of polymersomes. These are engineered amphiphilic block copolymers or vesicles, but in this circumstance they contain structures made up of one or more bilayers enclosing an aqueous center. In all conditions, the active load of molecules can be dissolved, dispersed, or chemically bound to nanocarriers. Their size is an important parameter for intracellular uptake. They offer better mechanical features and provide better structural and colloidal stability than liposomes in aqueous media [38]. Their flow properties, cellular uptake, and immune regulation depend upon their shape since their shape affects interaction with cells. As a result, being able to precisely rearrange a polymersome and comprehending the mechanisms underlying the various forms of interconversions is critical [39]. These polymers are amphiphilic in aqueous solvents and organized so that their hydrophilic head groups point toward the aqueous phase while their lipophilic tails are confined within the membrane’s core. The phase characteristic of a carrier in a solvent determines whether it can be built into a membrane [40].

Polymermerosomes are presently being studied for the delivery of different probes for imaging target tissues/organs and cytotoxic medications to canceous cells and also for gene therapy [41]. Zavvar and co-workers developed gadolinium-based quantum dots (QDs) of targeted PEG-PCL nanopolymersomes as a diagnostic agent and doxorubicin (DOX) as an anti-cancer agent. The controlled release of encapsulated DOX was revealed in the drug release study and the formulation was found to be stable in physiologic conditions. Enhancement in both toxicity and cellular uptake (*p* < 0.05) was revealed in MTT and flow cytometry results. Overall, the study demonstrated the theranostic application of targeted polymersomes with minimization of side effects [42]. Zhou et al., developed polymersomes based on photo-response for delivery of doxorubicin hydrochloride. The drug can be entrapped into their hydrophilic voids and their photo-responsive disintegration resulted in a controlled release of the drug. The cellular assay demonstrated their active targeting of folic acid and photo-activated release with higher cytotoxicity of HeLa cells. The study revealed the potential of polymersomes as effective and intelligent drug carriers [43].

Li et al., designed paclitaxel-loaded vesicles from matrix metalloproteinase (MMP)-cleavable peptide-linked triblock copolymer, polyethylene glycol-GPLGVRG-b-poly(ε-caprolactone)-b-poly(3-guanidinopropyl methacrylamide) that showed considerably improved cellular internalization effectiveness (~10 folds) in contrast to its pure form. The developed vesicles exhibited greater cytotoxicity against MMP-overexpressing HT1080 cells and multicellular spheroids. When MMP-2 was used for treatment it resulted in cleavage of stealthy PEG shell. The vesicles undergo morphological transformation into fused multicavity vesicles and small nanoparticles, accompanied by a redistribution of PGPMA segments with 76% exposed to the outside [44].

To increase chemical reaction efficacy, Li et al., created polymeric nanoreactors (NRs). They created therapeutic vesicular NRs (theraNR) that were loaded with glucose oxidase (GOD). When injected into normal tissues, the theraNR was inactive. The tumor acidity activates them precisely at the target location. They effectively destroy cancer cells by inhibiting their antioxidant capacity and perfusing tumors through a synergistic action [45].

Li et al., created a polymersome nanoreactor based on a block copolymer prodrug that may enable new cancer oxidation/chemotherapy via selective activation at tumor locations. GOD-loaded polymersome nanoreactors were created by optimizing block copolymers to self-accumulate into polymersomes in an aqueous solution for encapsulation of GOD. Through high tumor oxidative stress, oxidation/chemotherapy and released CPT medicines synergistically killed cancerous cells and inhibited tumor development [46]. 

By simply combining a couple of differently charged block copolymers (poly(amino acid)s and PEG) in an aqueous medium, Koide et al., produced a PICsome (a novel form of polymer vesicle along with a polyion complex (PIC) membrane) with a size range of up to 10 µm. Semipermeable characteristic of PICsomes membrane was discovered using spectral analysis and confocal laser scanning microscopic imaging. Even in the existence of serum proteins, the PICsomes maintained a high level of physiological stability, indicating that they may be used in medical applications such as therapeutic vehicles [47].

By incorporating stimuli-sensitive linkers into a crosslinking membrane network, Li et al., created responsive nano-reactors built on polyion complex vesicles. GOD could be protected by the fabricated ROS-responsive nano-reactor with self-improving catalytic glucose oxidation, which is attributed to stimuli-reactive vesicle development short of breakage and size-selective payload release pattern, to attain cytocidal role by oxidative stress induction and glucose starvation. Pyroptosis, an immunostimulatory type of cell demise, was produced by the GOD-loaded therapeutic nanoreactor [48].

### 2.4. Dendrimers

Dendrimers (accurately designed, highly-radial polymeric frameworks) were incorporated as promising drug nanocarriers, although some authors seem to be more satisfactory in their description as “polymer therapeutics” [49]. These are polymeric nanomaterials having 3-D macromolecule having branch-like shape, with less than 15 nm of size and coming out from a central portion [50,51].

Pillay et al., developed folic acid loaded dendrimer-functionalized selenium nanoparticles (FA-PAMAM-SeNPs) with size <150 nm and zeta potential of >25 mV. The developed formulation demonstrated greater cell viability (>85%) in contrast to placebo nanocomplexes (75%), confirming the importance of selenium in the developed formulation. The developed formulation resulted in greater overall transgene expression that showed their improved receptor-mediated cellular uptake [52].

Bartusik-Aebisher et al., developed a trastuzumab-dendrimer-fluorine DDS in which API was attached to dendrimers (fluorinated) via covalent bonding. They found that the potential of the developed formulation was more efficient than pure trastuzumab [53].

Mota et al., encapsulated I-buthionine sulfoximine in folate-targeted polyurea dendrimers. Initially, the burst release was observed that was expected in these delivery systems. After 1 h, about 60% of drug release was found, reaching a plateau after 3 h. After 24 h, 90% drug release was found [54].

Zhang et al., 2022 fabricated a third generation of poly(phosphorhydratzone) radical dendrimers and reported their action against the diagnosis of brain tumors [55]. It was also reported that the developed agent did not cause any toxicity to the cells.

Knauer et al., 2022 designed a polycationic phosphorous dendrimer-based approach for the delivery of siRNA to target Lyn [56].

### 2.5. Nanosized Hydrogels

Recently, the advancement of biological science managed novel developments in the area of in vitro cell culturing. Various novel innovations such as cells on chips/cell microarray need consistent supportive materials with decent biocompatibility, and cell adhesion and are simple to use [57]. Nanomaterial-based systemic drug vehicles need various properties particularly delivery of drugs to targeted sites. These parameters increase ligand conjugation and passive targeting, resulting in an improvement in active targeting. Hydrogel-medicated local DDSs directly deliver drug moieties at the target site without complications due to their implantation proximate to carcinoma tissues. It has been already found that a visible light-cured injectable glycol chitosan (GC) hydrogel-based local DDS can be employed for an ailment of various solid cancers [58]. Nanosized hydrogels developed by physically or chemically cross-linking polymer systems and commonly called nanogels can also be known as nanocarriers based on polymers [59]. Hydrogels may be custom-shaped and thickened, and their surfaces can be shaped using lithography methods. Furthermore, hydrogels can be fabricated with biomolecules to achieve unique characteristics [58].

Despite their solute permeability and controllable release features, hydrogels are efficient carriers for medicines, proteins, and others. Hydrogels made of chitosan can create multilayered structures, which is why they demonstrate utility in distributing bioactive molecules including insulin and growth factors, and in cell and tissue organization. They can be administered to the body using very simple, minimal stress procedures for the administration such as injection and both ocular and nasal administration [60].

### 2.6. Polymeric Cubosomes

Polymeric cubosomes are newly discovered inverse bicontinuous cubic mesophases. These are colloidal particles containing mesoporous block copolymers having well-defined reticulated pore networks. Kim et al., prepared polymer cubosomes using block copolymer which was synthesized by linking hydrophilic PEG and hydrophobic polyisoprene polymer blocks. The cubosomes were prepared via solution self-assembly of block copolymers [61]. The block copolymers with non-linear structures undergo self-assembly to form inverse mesophases in solution [62]. These are highly efficient nanoparticles that are developed from a lipidic cubic state and protected by an outer layer of polymer. The polymeric cubosomes are considered to have improved endurance and have a greater capacity to surround and embody hydrophobic anticancer agents due to their liquid crystalline membrane structure [63,64,65,66,67]. As a result of recent developments, applications involving drug delivery, membrane bioreactor, artificial cells and biosensors can be developed in vitro in both bulk and nanoparticle formulations [68].

**Figure 3:**
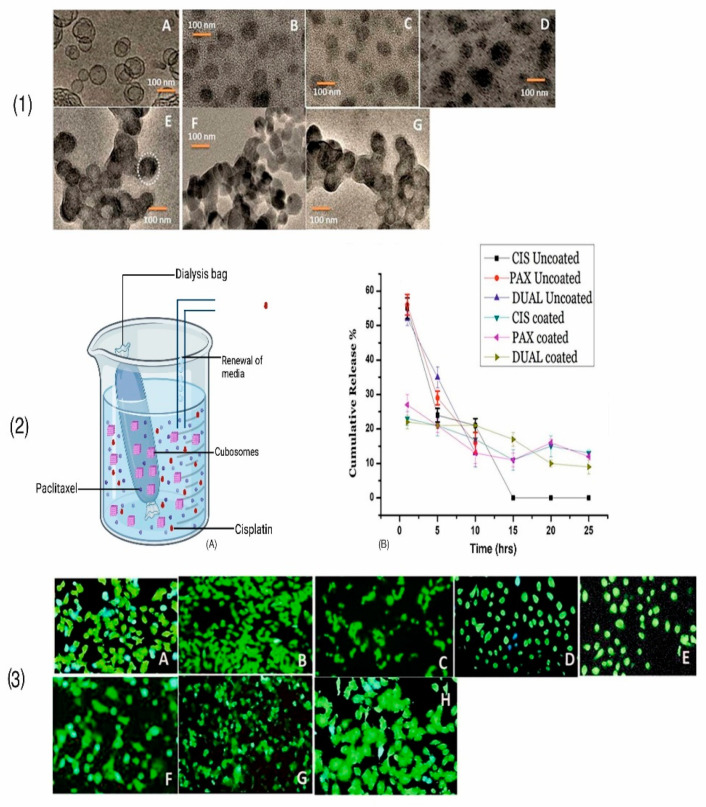
Illustration of (**1**) morphological analysis of (**A**) blank cubosomes, (**B**) uncoated CIS, (**C**) uncoated PAX, (**D**) uncoated DUAL, (**E**) coated CIS, (**F**) coated PAX and (**G**) coated DUAL cubosomes; (**2**) in vitro drug release studies (**A**) assembly and (**B**) comparative results of drug release; and (**3**) Cell viability studies on exposure of Human hepatoma HepG2 cells to (**A**) PBS blank cubosomes, (**B**) control cubosomes, (**C**) coated CIS, (**D**) uncoated CIS, (**E**) coated PAX, (**F**) uncoated PAX and (**G**) coated DUAL, (**H**) uncoated DUAL cubosomes. Reproduced from Zhang et al., 2020 [69] under creative commons CC BY license *©* 2020, The Author(s).

Zhang et al., developed the cubosomes loaded with a combination of anti-cancer drug cisplatin and paclitaxel for an effective targeting against cancer cells. In this work, a slow and sustained release was obtained by coating the cubosomes with poly-Ɛ-lysine. The coating prevented the initial burst of API resulting in a sustained drug release. TEM studies revealed very easily distinguishable spherical polyangular structures. Both initial reductions in burst discharge of drug and a slow, sustained discharge with time were revealed by in vitro studies. The cytotoxicity studies revealed the non-toxic behavior of cubosomes [69]. The morphological pattern, in vitro drug release, and cell viability investigations are shown in Figure 3. The impedance measurement and fluorescent imaging studies further confirmed their therapeutic efficiency against HeLa cells. Overall, the study affirms their significance as an effective nano-drug carrier.

Boge and co-workers developed the cubosomes for application in the delivery of peptide LL-37 for the ailment of skin infections. In the first strategy of development, the LL-37 was first added into a gel followed by dispersion into nanoparticles. The next strategy involved the adsorbing LL-37 onto preformed cubosomes. The last strategy consisted of the incorporation of LL-37 during their spontaneous formation in an ethanol/glycerol monooleate mixture. The pre-loaded cubosomes showed maximum efficiency in killing the bacteria [70].

## 3. Routes of Administration and Applications of Polymer Based Nanocarriers

The merging of polymer technology with pharmaceutical research resulted in a quantum leap in terms of innovation in designing and developing novel DDSs such as nanotechnology-based delivery systems. The application of nanopolymers in pharmaceutical and biomedical fields are swiftly growing, such as developing scaffold in tissue engineering, designing a drug delivery system, ophthalmology, dentistry, bone repair, implantation of medical devices, prosthesis, and many other medical fields. It is possible to obtain desirable properties in a pharmaceutical polymer by modifying its physical and chemical characteristics such as molecular weight, type, composition, co-polymerization, biomimetic, and co-processed excipient [71].

The discovery of the first synthetic drug delivery system sparked an interest in designing novel biodegradable polymers to replace the non-degradable polymer. The advent of bioadhesive polymer resulted in improved residence time and intimate contact between polymer and epithelial surface. Further advancements in polymer science led to the discovery of smart polymers that release the drug in presence of specific stimuli (physical, chemical, or biological) [72]. These nanopolymers were widely scrutinized based on their mechanism for controlled release of drugs and the understanding of their behavior in solid and liquid dosage forms, disperse systems, transdermal patches, implants, and many others [73]. There are numerous biodegradable and non-biodegradable polymers possessing different physicochemical characteristics used in pharmaceutical and biomedical fields (as shown in Table 1) [74,75,76,77,78,79,80,81,82,83,84].

Drug molecules (such as proteins, peptides, and DNA) are protected and stabilized by these polymers from environmental hazard degradation. Even though polymers have wide applications in the pharmaceutical industry, the criteria for proper selection and design of polymers is a challenging task and requires an in-depth understanding of its bulk properties (such as molecular weight, diffusion, and dissolution-controlled release based on solubility) and surface properties (such as surface energy, smoothness, hydrophilicity, lubricity, water sorption, and swellability) in addition to its physicochemical properties. The structure-activity relationship and polymeric matrices can be altered by changes in chemical composition and microstructural design [71]. The polymer-based nanocarriers are widely employed in various arenas for differenet routes that are discussed in brief below.

### 3.1. Applications of Polymeric Nanocarriers in Oral Drug Delivery

**Figure 4:**
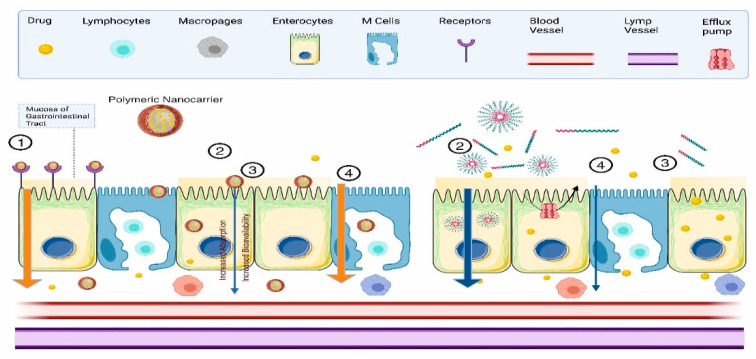
Illustration of mucoadhesion property of polymeric nanoparticles (**right**) and micelles (**left**) in gastrointestinal transit time modulation resulting in enhanced mucosal penetration and oral bioavailability. ① receptor-mediated endocytosis, ② transcellular transport, ③ paracellular transport, and ④ M cell-mediated transport.

The oral route has been explored as a non-invasive and preferred delivery route for numerous therapeutic agents. However, oral delivery suffers poor bioavailability and enzymatic instability [85,86,87]. Modulation of gastrointestinal transit time is a challenge that can be overcome by increasing the residence time of drugs within the gastrointestinal tract. Polymeric nanocarriers emerged as prominent oral delivery vehicles owing to their interesting bioavailability, superior drug entrapment, control release, and minimal toxicity. The improved oral bioavailability of polymeric nanocarriers is due to the enhanced mucoadhesion property of polymer that sticks to the mucosa of the gastrointestinal tract leading to prolonged gastro-intestinal transit time and thereby enhancing mucosal penetration (as shown in Figure 4).

Polymeric materials being biocompatible, biodegradable, non-immunogenic, and functional that offer numerous advantages for drug delivery such as active drug targeting via surface functionalization and controlled release in the body [88]. Polymers-based nanocarriers have been extensively explored as vehicles for oral delivery of various peptides, genes, medicaments, proteins, siRNA, probiotics and vaccines as enlisted in Table 2 [89,90,91,92,93,94,95,96,97,98,99,100,101,102,103,104,105,106,107,108,109,110]. Polylactic acid (PLA), poly-glycolic acid (PGA), polycyanoacrylates (PCA), polyethyleneimine, and polycaprolactone are the main synthetic polymers widely employed for oral drug delivery. Chitosan, dextran, gelatin, alginate, and agar among which chitosan is most widely used owing to its mucoadhesive, biocompatible, and non-toxic properties [100].

### 3.2. Applications of Polymeric Nanocarriers in Ocular Drug Delivery

**Figure 5:**
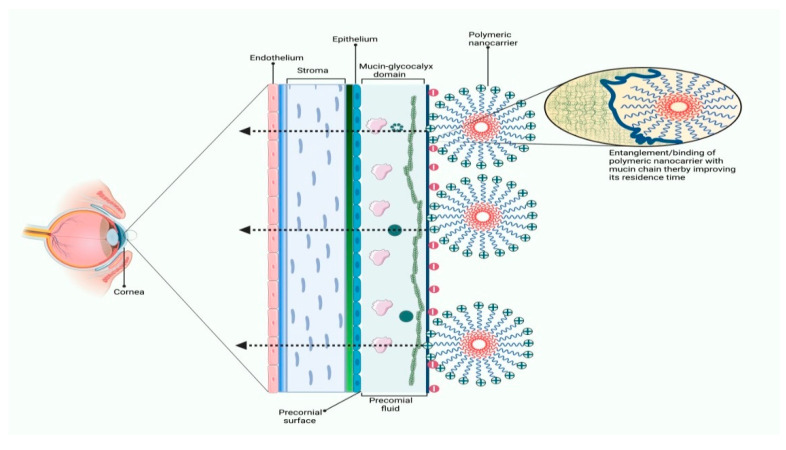
Illustration of ionic interaction of polymeric nanocarriers with corneal glycocalyx domain resulting in increased ocular surface contact and improved drug penetration.

Several efforts have been made in ocular drug delivery to increase corneal assimilation by improving the drug residence time with the ocular surface through bioadhesive polymers [111]. The precorneal fluid contains a mucin-glycocalyx domain which is bound to the corneal surface and serves as a substrate for adequate binding of polymeric chains present in ocular formulation through non-covalent bonding resulting in prolonged drug contact time with the ocular surface and thereby improved bioavailability (as demonstrated in Figure 5). The widely used polymers for ocular drug delivery include chitosan, gelatin, alginate, synthetic poly-alkyl cyanoacrylate, poly-ε-caprolactone, poly-lactic acid, poly-lactic-co-glycolic acid, and polystyrene [112]. The biological properties of the polymer such as biodegradability, non-toxicity, biocompatibility, and mucoadhesiveness resulted in the development of various colloidal vehicles for ocular routes such as polymeric nanoparticles and polymeric micelles [113]. The positive surface charge of polymeric nanocarriers allows prolonged contact time of API on the ocular surface due to increased interaction with glycoprotein of cornea and conjunctiva leading to the formation of a precorneal depot that is responsible for the prolonged release of API.

Sharma et al., formulated polymeric nanoparticles using bioadhesive positively charged Eudragit^®^ RS100 and Eudragit^®^ RL100 which are official by USFDA as excipients for controlled drug delivery. The presence of a positive charge on nanoparticles resulted in ionic interaction with anionic mucin existing in the mucus layer of tear film leading to sustained release of drug and improved ocular penetration [114]. Mahor et al. fabricated moxifloxacin-loaded polymeric nanoparticles using positively charged gelatin for its effective ocular delivery and controlled release in the corneal eye layer [115]. Mittal et al. utilized chitosan, a polycationic bioadhesive polymer, to develop timolol maleate-loaded polymeric nanoparticles. The polymeric nanoparticles exhibited significant bioadhesive strength due to the existence of polymeric chains and polar functional groups in chitosan. The in vitro experiment displayed burst release (27.18% in 1 h) and then sustained release (90.55%) up to 24 h. The ex vivo transcorneal permeation investigation exhibited appreciable corneal penetration of timolol from polymeric nanoparticles compared to conventional eye drops attributable to the penetration-enhancing property of chitosan [116]. Table 3 compiles significant reports on the ocular delivery of polymeric nanocarriers [117,118,119,120,121,122,123,124,125,126,127,128,129,130,131,132,133,134,135,136,137,138,139,140,141,142,143,144,145,146,147,148].

### 3.3. Applications of Polymeric Nanocarriers in Parenteral Drug Delivery

**Figure 6:**
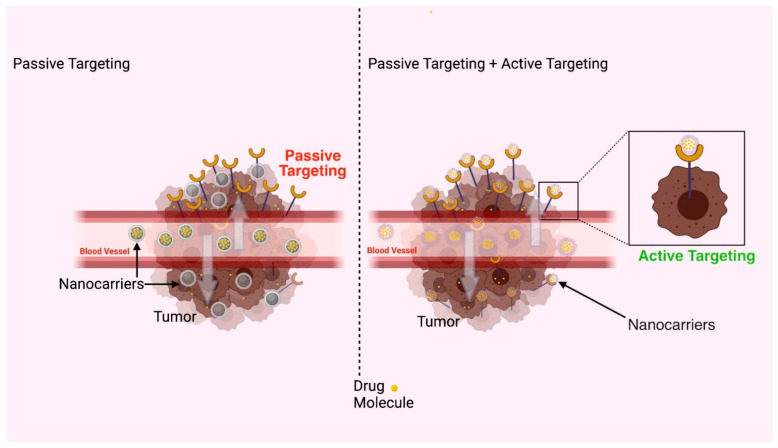
Graphical representation of active and passive targeting of drugs via polymeric nanocarriers following parenteral administration.

This is the preferred route for delivery of active ingredients having a narrow therapeutic index and poor bioavailability. However, intramuscular and subcutaneous injection dosage form suffers rapid drug elimination. Continuous *IV* infusion is one of the approaches for maintaining constant drug delivery which also avoids hepatic metabolism but the patient has to be hospitalized. Among methodologies to overcome traditional medical treatment, simulation of *IV* infusion by use of parenteral controlled release formulations has gained interest. Figure 6 depicts sustained and targeted delivery of drugs via polymeric nanocarriers following parenteral administration [148].

Active targeting can be achieved at different levels depending on the extent of penetration; it may occur at the organ, cell, or subcellular level. It is important to note that in solid tumors, even active targeting processes begin with the passive accumulation of the DDS in the tumor tissue, so any actively targeted carrier must satisfy the basic requirements outlined for passively targeted systems: the polymer/nanoparticle should be biocompatible, and the system must be stable in circulation, long-circulating, and of a size that permits efficient extravasation and accumulation in tumors. In addition, actively targeted systems are often more effective in general than passively targeted alternatives [149].

Intravenous administration of biodegradable polymeric nanocarriers has been effectively employed for controlling and targeting drugs to a specific site of action. In particular, biodegradable nanocarriers formulated from PLGA have been extensively studied for sustained and targeted delivery of numerous agents [150]. It is a copolymer synthesized by co-polymerization of two different monomeric units, the cyclic dimers of glycolic acid and lactic acid, linked through an ester linkage. PLGA has been approved by FDA and European Medicine Agency for usage in parenteral DDSs due to its biocompatibility and biodegradability. Several forms of controlled DDSs have been studied among which polymeric nanocarriers have gained attention for diagnostics, prognostics, controlled and sustained delivery of protein, peptide, pDNA, and other therapeutic agents owing to their biocompatibility, non-immunogenicity, and several others are enlisted in Table 4 [151,152,153,154,155,156,157,158,159,160,161]. Natural and synthetic polymers have been extensively investigated as carriers for several anticancer drugs. Conjugation of drugs with these polymeric carriers increases their circulation time in the blood leading to passive gathering in tumor tissues due to the enhanced permeability and retention (EPR) effect [140,141].

### 3.4. Applications of Polymeric Nanocarriers in Topical Drug Delivery

Polymer-based nanocarriers have been extensively explored as topical formulations to improve cutaneous delivery owing to their physicochemical attributes which prevent degradation or denaturation of unstable drugs, reduce their toxic side effects through controlled release, and enhance their cutaneous penetration across the biological membrane more precisely skin barrier due to their ability to increase the concentration gradient [162]. Polymers serve an important role in the preparation of polymeric nanocarriers which might be natural or synthetic. The extensively used natural polymers are chitosan, alginate, gelatin, and albumin. Synthetic polymers include polylactides, PVA, poly(acrylic acid), polyacrylamide, PEG, and several others. These polymers have gained attention as a substrate for colloidal vehicles due to their blood stability, non-toxicity, non-thrombogenic, non-immunogenic, and non-inflammatory characteristics [163,164].

Natural polymers tend to develop hydrogels which render them suitable carriers for peptides, oligonucleotides, proteins, and water-soluble APIs for improving the absorption and targeted drug delivery of polymeric nanocarriers for topical administration. Chitosan-based nanocarriers have been widely used among the natural polymers for topical delivery. An N-deacetylated derivative of chitin, chitosan is a cationic polymer that is made up of glucosamine units. Its additional anti-oxidant, anti-inflammatory, and anti-microbial features make it an appropriate carrier for therapeutic delivery. Table 5 reports a compilation of polymeric nanocarriers for topical delivery of drugs, peptides, and genes [148,149,150,151,152,153,154,155,156,157,158,159,160,161,162,163,164,165].

## 4. Practical and Regulatory Consideration

Nanomedicine is a new area integrating nanotechnology with pharmaceutical and biomedical sciences, intending for the development of medicines with improved safety and toxicological profiles [171]. In drug formulation, nanoparticles can impart several physical and biological advantages such as improved pharmacokinetic profile, enhanced tissue/organ selectivity, and decreased toxicity as compared to conventional medicines [172]. FDA governs the pre-market approval process of conventional drugs, biologics, and medical devices, and likewise, nanomedicines are also subject to a normal range of pre-clinical and clinical validation. Based on available literature and the Centre for Drug Evaluation and Research (CDER), the no. of clinical trials involving nanomedicines has increased three-fold and the utility of nanomaterials in these products has incredibly risen in the past two decades [173]. Based on previously reported results, liposomes account for the largest proportion of therapeutic nanoproducts with over 33%, followed by nanocrystals and emulsions which accounts for 23% and 14%, respectively. Nine percent of products used include polymer ion complexes while micelles have a 6% share. The studies also show that specific nanostructures (quantum dots, carbon nanotubes, graphene) also have a small percentage of shares in these products [174].

In nanomedicine, polymeric nanoparticles serve two purposes (i.e., controlled release of drug-using biodegradable polymers and improved half-life and bioavailability by forming drug-polymer conjugates) [175]. Copaxone and Neulasta are two polymeric drugs out of the top 10 bestselling drugs in the US in 2013 [176]. There are several polymeric immobilized nanomedicines as shown in Table 6 that are approved by USFDA [177,178,179,180,181,182,183,184].

The composition and structure of polymeric micelles can be precisely modified to achieve specific release characteristics and drug loading. The only approved polymeric micelle is a traditional formulation of oestradiol (Estrasorb™). The use of modern co-block polymers led to the development of polymeric micelles at a lower critical micellar concentration with high stability [185]. Few polymeric micelles formulations that are in late-stage clinical trials, especially *i*/*v* administered formulations are shown in Table 7 [186].

The various patented reported polymeric nanocarriers intended for oral, ocular, topical, and parenteral administration are enlisted in Table 8 [187].

### 4.1. Toxicity Issues

Most of the polymeric nanocarriers are generally regarded as safe owing to the interesting physicochemical characteristics of the polymer which render them biocompatible and biodegradable. Copolymers PLA and PLGA acid are the most extensively employed biomaterials for polymeric nanocarriers, that undergo hydrolytic degradation within the body leading to the generation of endogenous lactic acid and glycolic acid monomers which are metabolized via Kreb’s cycle leading to minimal systemic toxicity [188]. However, various factors could affect the toxicity of the polymeric nanocarriers not limited to their surface chemistry, size, and concentration as reported by Ahlawat and Henriquez [189]. The size of the polymeric nanocarriers significantly affects the cellular uptake and hence causes toxicity. Enhanced cellular uptake of polymeric nanocarriers leads to increased interaction with intracellular components which triggers the production of free radicals, cytokines, and reactive oxygen species (ROS) causing organelle destruction and finally cell lysis that leads to toxicity issues [190,191].

Grabowski and Hillaireau studied the toxicity of surface-mediated polymeric nanoparticles on human-like THP-1 macrophages wherein cell mitochondrial activity, apoptosis detection, ROS, and pro-inflammatory cytokines production were chosen as toxicity criteria. Positively, negatively and neutral PLGA nanoparticles were fabricated with chitosan, poloxamer 188, and PVA stabilizers. The stabilizer-free PLGA nanoparticles did not exhibit any sign of toxicity at or above a therapeutic concentration of 0.1 mg/mL. However, PLGA nanoparticles prepared using stabilizers exhibited cytotoxicity above the therapeutic level of 0.1 mg/mL, thereby confirming the safety of the polymeric nanocarriers and the toxicological contribution of stabilizers used in the polymeric nanocarrier formulation [192].

Rejinold and Muthunarayanan reported non-toxicity of the chitosan-g-poly-N-vinyl caprolactone nanoparticles in an array of cell lines at 100–1000 µg/mL [193]. Similarly, Dandekar and Dhumal reported the cellular safety of the Eudragit^®^ S100 polymeric nanoparticles through genotoxicity studies [194]. Singh and Ramarao examined the concentration-dependent toxicity of PLGA, PLA, poly-ϵ-caprolactone, and poly-lactide-caprolactone nanoparticles using macrophage (RAW 264.6), hepatocytes (Hep G2), lung (A549), kidney (A498) and neuronal epithelial (Neuro A2) [180]. Polymeric nanoparticles did not display toxicity at 100 µg/mL concentration as marked by no change in cell viability. However, a reduction in cell viability was detected at 300–1000 µg/mL concentration after 72 h incubation of polymeric nanoparticles with cell lines.

### 4.2. Effects of the Route of Administration on Bioavailability

Polymeric nanocarriers have become a prominent field of research in the arena of drug delivery since they can improve pharmacokinetic and bioavailability that results in effectiveness of various drugs, enhanced hydrophilicity, reduction of interaction with plasma and cellular proteins, and greater desposition of drug at target sites [195]. Oral, intravenous, intramuscular, intranasal, intradermal, and transdermal administration are the foremost delivery routes for drugs. Other routes, such as ocular delivery, have also been developed for localized and targeted drug delivery, ocular route is employed that declines unwanted systemic side effects. Specific barriers are encountered by each route of administration route. Roughly, solubility and permeability are two major issues related to drug substances that are responsible for bioavailability upon administration [196]. Improved stability and bioavailability of therapeutic agents coupled with extended drug release profile and possibility to embed numerous drugs makes suitable candidate for pharmaceutical products [197].

Various factors govern the assimilation of the drug such as drug solubility, mucosal permeability, and stability in the GI tract milieu. Efforts are ongoing to tackle these issues have focused on physicochemical, biochemical, biological, and metabolic barriers that are responsible for limited assimilation. Different pharmaceutical technologies and DDSs such as nanocarriers, micelles, cyclodextrins, and lipid-based carriers have been discovered for improving the assimilation of a drug.

GI tract leads to experience a varied range of pH, enzymatic degradation and low permeability across the membrane. Polymeric NPs may be an auspicious strategy that can improve stability to crossing the mucus barrier or interact with a mucus layer and increase residence and contact time [198].

Oral, aerosol, and nasal vaccination is a novel immunization technique that employ nanocarriers to administer vaccine. Therapeutic agents and vaccines with lower penetration potential can be administer via transmucosal route while maintaining their biological activity. Nowdays, nanocarriers for transmucosal delivery are gaining more attention since delivery via oral mucosa helps evade first-pass metabolism, improves bioavailability and acts as a means of rapid drug transport to the systemic circulation. The oral transmucosal vaccine delivery by nanocarriers is the most upcoming innovation in efficient vaccine delivery [199,200].

## 5. Future Prospects

Over the past few decades, polymer science has evolved to mediate safe and efficient drug delivery for the ailment of several health conditions. Over the years, the usage of polymers has been limited to oral delivery, but critical advances in polymer science such as the development of biodegradable nanosized carriers have accelerated their use in parenteral formulations. Further innovations in the application of polymers have led to a more sophisticated approach, thereby improving the pharmacokinetics, decreased toxicity, stealth effect, cell or organ-specific targeting, and controlled release of the drugs; these innovatioms have made it possible to administer two or more drugs or imaging/diagnostic agents within the same drug delivery system, leading to more effective therapy [198,201,202]. Due to the development of novel polymers with highly complex structures and functions, its scope has widened in the field of nanoformulation, particularly in imaging and theranostic applications [203]. The recent trend in polymer science is leaping from the traditional linear polymer to the complex hyperbranched or hybrid polymers.

New technologies and advances in the synthesis and manufacturing process such as 3-D printing, supercritical fluid technology, and nanoengineering are revolutionizing polymer-based drug delivery systems [204]. Scale-up in the field of nanotechnology has always been troublesome due to non-reproducibility between batches and future evolution is expected from the introduction of new types of polymers also known as smart polymers (stimuli-responsive, enzyme responsive) that fulfill requirements to be used as a constituent in drug delivery systems [205]. Nevertheless, future development should progress towards understanding the interaction of polymers or polymercarriers with the biological system. Most studies carried out in the present scenario neglect the biocompatibility studies that fail new devices at the later stage of development. Adequate animal models and in vitro studies can lead to a breakthrough in polymer science as safe and effective platforms for drug delivery [206].

Nanomedicine has evolved over the last several decades from biologically inert substances to increasingly intelligent systems aiming at improving in vivo functioning. But, we must acknowledge that most systems depend on logical explanations-including some over-explanation rather than conclusive proof, which is a pivotal event in nanomedicine’s progress. The importance of investigating nano-bio interactions and desired functioning at the molecular, cellular, and tissue levels is often ignored [34].

An overall change in global markets, technological innovations, and data on the pathophysiological and cell-based mechanisms should be considered that can outline the future progress for polymers in drug delivery.

## 6. Conclusions

Polymeric nanocarriers have a great potential for clinical applications. They are expected to perform both diagnostic and therapeutic functions due to their interesting physiochemical characteristics which offer numerous advantages such as biocompatibility, biodegradation, non-immunogenicity, non-inflammatory, low toxicity, prolonged drug release, and active site targeting, rendering them safe for therapeutic applications at a certain concentration. At present, few polymeric systems can be employed for diagnostic and therapeutic applications. Among nanomaterials, polymeric nanocarriers have been the exceedingly explored delivery vehicles due to their wide range of solubility (hydrophilic and lipophilic surfaces) and self-assembling properties. They offer solubilization of hydrophobic or poorly soluble molecules, sustained drug release, and protection to encapsulated drugs from degradation and metabolism. The polymeric nanocarriers are considered as the best ones due to their good drug loading capacities, biodegradability, and stimuli-responsive control.

Polymeric nanocarriers are safe for the parenteral route of administration than their surfactant counterparts. They are kinetically stable and hence dissociate very slowly in blood, resulting in extended circulation time. Recently biopolymeric nanocarriers have immerged as storms which are developed using polymers obtained from natural sources. These biopolymeric nanocarriers offer better biodegradability, biocompatibility and could be the next generation nanocarriers for drug delivery. In the literature, there are several studies for developing new generation polymeric nanocarriers to obtain future smart and multifunctional nanomedicine. 

Polymeric nanocarriers have many challenges for a carrier system such as biocompatibility, biodistribution, side effects, and biological barriers. Their biodegradability and biocompatibility profiles should be critically investigated before utilization in human clinical trials. One major concern that requires immediate attention is the regulatory concerns regarding the approval of polymer-based carriers since establishing new guidelines and policies seem to be in the initial stages of development. New criteria for manufacturing, testing, and storage of these nanosized polymeric materials should be defined by the regulatory agencies [207]. So, their manufacturing and regulation have challenges for their further development in various fields.

## Figures and Tables

**Table 1:** Applications of nanopolymers in pharmaceutical and clinical medicine.

Classification	Name of Polymers	Applications of Polymer	Ref.
Natural polymers			
Protein based	Soy, whey, collagen, gelatin	Gene delivery, nanoparticles	[74]
Polysaccharides	Alginate, pectin, guar gum, chitosan, chondroitin, heparin, hyaluronic acid, cyclodextrins	Binding and film coating agents in tablets, mucoadhesive, controlled release of drugs	[75]
Synthetic polymers			
Biodegradable			
Polyamides	Polyamino acids, poly(iminocarbonates) and their copolymers	Sutures, catheters for angioplasty, controlled and sustained drug delivery	[76]
Cellulose	Carboxy methylcellulose, ethyl cellulose, cellulose acetate, HPMC	Binder, coating, emulsifying, disintegrants in tablets and capsules	[77]
Polyanhydrides	Poly(adipic) acid, poly(sebacic) acid, and their copolymers	Controlled release coatings, medical implants	[78]
Polyesters	Poly (lactic acid), poly (glycolic acid), poly(dioxanes) and their copolymers	Protein delivery, dialysis membrane	[79]
Others	Polyurethane, polyortho esters, poly(cyano) acrylates, polystyrenes	Sutures, stents, drug delivery devices, dialysis media	
Non-biodegradable			
Silicones	Colloidal silica, polydimethylsiloxane	Therapeutic devices, implants, medical grade adhesive for transdermal delivery	[82]
Acrylic polymers	Polymethacrylates, polyhydroxy (ethyl acrylates)	Thermo-gelling acrylamide derivatives, its balance of hydrogen bonding, and hydrophobic association changes with temperature (smart polymers), film-forming agent	[83]
Others	PVP, poloxamers, ethyl vinyl acetate	Tablet granulation, plasma replacement	[84]

**Table 2:** Various nanopolymers employed as nanocarriers for oral delivery of drugs, peptides, and genes.

Polymer	Delivery System	Therapeutic Molecule	Summary	Ref.
PLGA	NPs	Coumarin-6	Surface modification of PLGA nanoparticles with vitamin E TPGS notably improved the cellular uptake to promote oral chemotherapy.	[89]
Carboxylated chitosan	NPs	Bovine serum albumin	Chitosan grafted nanoparticles showed increased intestinal absorption due to higher mucoadhesion, cellular uptake, and systemic biodistribution after oral administration.	[90]
Polystyrene	NPs	NA	Vitamin E TPGS modified polystyrene nanoparticles evidenced increased cellular uptake by *Caco-2* and MDCK cells employed as an in vitro model for gastro-intestinal and blood-brain barrier respectively. In vivo investigation displayed that vitamin E TPGS coated nanoparticles (<200 nm) can escape RES and thus enhance half-life.	[91]
PLGA and alginate stabilized chitosan	NPs	Clotrimazole and econazole	Oral administration of polymeric nanoparticle encapsulated drugs showed controlled drug delivery for 5–6 days compared to unencapsulated drugs which were cleared within 3–4 h following oral or IV administration. Bioavailability of drugs was remarkably improved and was detected in the lung, liver, and spleen tissues till 6–8 days compared to free drugs which were cleared by 12 h.	[92]
PLGA and polymethylmethacrylate	NPs	Enalaprilat	The in vitro study across rat jejunum showed that the apparent permeability coefficient of enalaprilat-loaded PLGA nanoparticles was not significantly improved compared to the enalaprilat solution.	[93]
PLG	NPs	Rifampicin, isoniazid and pyrazinamide	Oral administration of drug-loaded nanoparticles in mice showed prolonged blood circulation for up to 6 days for rifampicin and up to 9 days for isoniazid and pyrazinamide. The therapeutic concentration of the drug in the tissues was maintained for 9–11 days.	[94]
N-isopropyl acrylamide, methylmethacrylate and acrylic acid in 60:20:20	NPs	Rapamycin	Significant blood levels of rapamycin were observed within 30 min after oral administration of rapamycin-loaded polymeric nanoparticles and continue to be detected in bloodstream up to 24 h.	[88]
PLGA	NPs	Paclitaxel	Orally administered paclitaxel-loaded PLGA nanoparticles displayed a 5-fold increase in apparent permeability across *Caco-2* cells, employed as in vitro model compared to free paclitaxel. Surface functionalization with folic acid further increased the transport of nanoparticles by 8-fold.	[95]
Eudragit^®^ RS	NPs	Insulin	Oral delivery of polymeric nanoparticles preserved the biological activity of encapsulated insulin and showed increased serum insulin level for a prolonged period due to the mucoadhesive property of polycationic polymer (Eudragit^®^ RS) facilitating insulin intestinal uptake.	[96]
PLGA	NPs	Doxorubicin	Time and concentration-dependent increase in cellular uptake of doxorubicin-loaded polymeric nanoparticles was observed across *Caco-2* cells compared to doxorubicin solution. Orally administered nanoparticles displayed reduced cardiotoxicity compared with the intravenously injected free drug solution.	[97]
Chitosan	NPs	Alprazolam	The cationic chitosan polymer was intercomplexed with anionic egg albumin and stabilized with PEG 400 to develop nanoparticles which demonstrated sustained drug delivery up to 24 h.	[98]
Trimethyl chitosan	NPs	TNF-α siRNA	Following oral administration mannose functionalized chitosan-cysteine conjugate nanoparticles enhanced siRNA stability in physiological fluid and promoted its transport across intestinal epithelium leading to siRNA uptake by macrophages through endocytosis and cytoplasmic siRNA release.	[99]
PCL, PLGA and Eudrajit^®^ RS and RL	NPs	Heparin	Orally administered polymeric nano-particulate heparin showed prolonged anti-Xa activity compared to the heparin solution injected intravenously. A 2-fold increase in activated partial thromboplastin time was reported.	[100]
Poly-(DL-lactide-co-glycolide)	NPs	*pDNA* (alkaline phosphatase, a reporter gene)	*pDNA* loaded in PLGA polymer particles showed sustained release of pDNA while maintaining its structural and functional integrity for over a month.	[101]
PLGA	NPs	Tamoxifen	The oral bioavailability of tamoxifen was increased by 3.84 times compared to free tamoxifen citrate and 11.19 times compared to free tamoxifen when formulated in PLGA nanoparticles. Histopathological studies evidenced the low toxicity of tamoxifen encapsulated nanoparticles compared to free drug.	[102]
Chitosan	NPs	Antigen (bovine serum albumin)	Antigen-loaded chitosan nanoparticles; surface engineered with *Ulex europaeus agglutinin* lectin displayed efficient systemic and mucosal immune responses.	[103]
PEG-poly(lactide) diblock copolymers	Micelles	Cyclosporine A	Enhanced stability and intestinal absorption of cyclosporine A-loaded polymeric micelles were reported compared to commercial tablet formulation of cyclosporine A.	[104]
Chitosan	NPs	Rifampicin	pH-dependent drug release (75%) at simulated intestinal pH over a period of 24 h	[105]
Poly lactic acid-co-PEG	Micelles	Fenofibrate	Micelles having a size range of 158 to 249 nm were prepared as a carrier for oral administration.	[106]
Chitosan	Micelles	Paclitaxel	3.80-fold enhanced bioavailability of paclitaxel micelles compared to Taxol^®^	[107]
Polyvinyl caprolactam-polyvinyl acetate-PEG graft copolymer	Micelles	Silybin	Significant increase in absorption of Silybin following oral administration of drug-loaded polymeric micelles in rats.	[108]
Carbomer 934 and poloxamer P 407	Micelles	Deferoxamine mesylate	Polymeric micelle exhibited 2.5 times increased drug permeation across intestine compared to control.	[109]
N-naphthyl-N,O-succinyl chitosan	Micelles	Curcumin	pH-responsive polymeric micelles exhibited a significantly increased amount of drug release in simulated colonic fluid compared to free drug.	[110]

NPs, nanoparticles; NA, not applicable.

**Table 3:** A cross-section report on polymers employed as nanocarriers for ocular drug delivery.

Polymer	Delivery System	Therapeutic Molecule	Summary	Ref.
Eudragit^®^ RL and RS (50:50)	NPs	Gatifloxacin	Prolonged-release rate and antimicrobial activity.	[117]
PLGA and PLGA–PEG	NPs	Melatonin	PLGA–poly (ethylene glycol) loaded melanin displayed prolonged pharmacological effect (reduced intraocular pressure) up to 8 h.	[118]
Chitosan-alginate	NPs	Daptomycin	In vitro ocular permeability study of daptomycin-loaded chitosan-alginate nanoparticles showed increased epithelial retention compared to free drug.	[119]
PLGAwith Eudragit^®^RL or coated with Carbopol^®^	NPs	Cyclosporin A	Polymeric nanoparticles displayed biphasic release i.e., initial burst followed by slow drug release up to 24 h. PLGA with Eudragit^®^RL showed the highest degree of cellular uptake, tear film concentration, and ocular bioavailability.	[120]
PLGA	NPs	Pilocarpine	Chitosan-coated PLGA nanoparticles of pilocarpine displayed prolonged residence time after topical ocular application.	[121]
PLGA	NPs	Moxifloxacin	PLGA nanoparticles loaded with moxifloxacin showed higher drug permeation compared to conventional eye drops. Sustained drug release was observed up to 24 h, thereby could avoid frequent administration of dosage.	[122]
Gelatin	NPs	Moxifloxacin	Moxifloxacin-loaded gelatin nanoparticles exhibited burst release in the first hour followed by controlled release up to 12 h in an in vitro experiment.	[115]
Sodium alginate	NPs	Brimonidine tartrate	In vivo experiment in albino rats displayed prolonged drug release up to 8 h following topical application of brimonidine tartrate loaded gelatin nanoparticles.	[123]
Chitosan	NPs	Betaxolol hydrochloride	In vitro drug release study showed an initial burst followed by sustained release up to 12 h. This could be due to mucoadhesiveness of chitosan leading to improved pre-corneal residence time and hence corneal permeability.	[124]
Eudragit^®^ RS 100	NPs	Cyclosporin A	In vitro experiment of cyclosporin A loaded positively charged Eudragit^®^ RS 100 nanoparticles displayed extended drug release. In vivo results showed prolonged residence time of drug and polymeric nanoparticles in vitreous humor.	[125]
Poly-ε-caprolactone, 2-hydroxy ethyl methacrylate, PEG diacrylate	NPs	Loteprednol	A drug release study of hydrogel-embedded polymeric nanoparticles showed extended release for up to 12 days.	[126]
Chitosan-sodium alginate	NPs	Gatifloxacin	Mucoadhesive polymeric nanoparticles showed fast release during 1 h followed by gradual release up to 24 h.	[105]
Poly(beta-amino esters)	NPs	Genes	Polymeric nanoparticles showed transfection efficiency of up to 85% for human reticuloendothelial cells and up to 65% for human umbilical vein endothelial cells.	[127]
Poly(D,L-lactide co-glycolide) (PLGA)	NPs	Loteprednol etabonate	Ex vivo trans corneal permeation study across goat cornea revealed an improved permeation profile of formulated drug product compared to the plain dug owing to an increased residence time of PLGA nanoparticles.	[128]
Chitosan	NPs	Ornidazole	In vitro drug release study revealed initial burst release followed by gradual release up to 24 h of the ornidazole-loaded mucodhesive chitosan nanoparticles.	[129]
Chitosan	NPs	Naringenin	In vitro study of naringenin-loaded chitosan nanoparticles revealed a moderate sustained-release effect. In vivo experiment exhibited prolonged residence time of polymeric nanoparticles compared to naringenin suspension which could be accountable for its improved bioavailability in aqueous humor.	[130]
Methoxy poly(ethylene glycol)–poly(ɛ-caprolactone)	Micelles	Not applicable	In vitro studies of polymeric micelles did not show any cytotoxicity against human corneal epithelial cells, human lens epithelial cells, and retinal pigment epithelial cells at micellar concentrations of 0–2 mg/mL.	[131]
Eudragit^®^ RS100	NPs	Vancomycin	Prolonged residence time and C_max_ of vancomycin-loaded polymeric nanoparticle was observed resulting in a more than two-fold increment in bioavailability (AUC_0.25–24_) over control group.	[132]
Poly(dl-lactide-*co*-glycolide) (PLGA)	NPs	Sparfloxacin	In vitro release study exhibited an extended drug release profile. Gamma scintigraphy study in albino rabbits showed prolonged precorneal retention of the radiolabelled sparfloxacin-loaded polymeric nanoparticles compared to the marketed formulation. The formulation displayed non-irritant properties in the Hen egg test-chorioallantoic membrane test.	[133]
Poly(ethylene oxide)-poly(propylene oxide)	Micelles	Lornoxicam	Confocal laser studies evidenced the appreciable corneal penetrating power of the polymeric micelles.	[134]
Eudragit^®^ RS100 and Eudragit^®^ RL100	NPs	Cloricromene	In vitro studies showed modified release of drug from the polymer matrix.	[135]
Poly(D,L-lactide-co-glycolide)	Nanospheres	Flurbiprofen	Polymeric nanospheres showed a two-fold increment in drug permeation compared to commercial eye drops formulation in an ex vivo experiment.	[136]
Eudragit^®^ RL100	NPs	Aceclofenac	In vitro transcorneal permeability study across excised goat cornea revealed a 2-fold increment in drug permeation from polymeric nanoparticles compared to aqueous drug solution. No signs of corneal damage were reported.	[137]
PLGA and chitosan	NPs	Fluocinolone acetonide	Polymeric nanoparticles showed good mucoadhesion characteristics and exhibited rapid and extended drug delivery to the eye evidenced in the pharmacokinetic experiment.	[138]
Methylated β-cyclodextrin	NPs	Fenofibrate	Polymeric NPs resulted in low cytotoxicity, low hemolytic potential, and moderately irritable to the eyes.	[139]
Zein and hyaluronic acid	NPs	Ciprofloxacin	The developed NPs were biocompatible, had high %EE, and prolonged release of the drug. These can be employed for the treatment of conjunctivitis.	[140]
Chitosan	NPs	Meloxicam	Polymeric nanoparticles showed sustained drug release behavior and improved permeation through the cornea.	[141]
Poly(L-lactide)-b-poly(methacrylic acid-co-3-acrylamidophenylboronic acid)	Polymeric micelles	Cyclosporin A	The developed micelles resulted in low cytotoxicity, reduction of dose, and improve bioavailability of the therapeutic molecule.	[142]
Soluplus	Polymeric micelles	Ibuprofen, idebenone, and miconazole	The developed nanomicelles have potential applications in ocular delivery.	[143]
PEGylated fatty acid-block-poly(ε-caprolactone)	Polymeric micelles	Cyclosporin A	The developed nanomicelles showed sustained drug release behavior and can be employed for ocular drug delivery.	[144]
PEG-b-PLA	Polymeric micelles	Triamcinolone acetonide	The developed micelles enhanced anti-inflammatory action.	[145]
PLGA	Polymeric nanospheres	Lactoferrin	In vitro and in vivo investigations revealed a significant increment in residence time of developed formulation on the eye surface.	[146]
PLGA-PEG	Polymeric nanospheres	Pioglitazone	The ex vivo investigations of developed nanospheres revealed that permeation and retention via sclera were greater than corneal and non-irritant for the eye.	[147]

NPs-Nanoparticles, PLGA—Poly(dl-lactide-co-glycolide), PEG—Poly(ethylene glycol), PLA—Polylactic acid.

**Table 4:** A summary of reports on polymers employed as nanocarriers for parenteral delivery of drugs, peptides, and genes.

Polymer	Delivery System	Therapeutic Molecule	Summary	Ref.
PLGA	NPs	*pDNA* (alkaline phosphatase, a reporter gene)	*pDNA* loaded in PLGA particles showed sustained release of pDNA while maintaining its structural and functional integrity for over a month.	[101]
PCL	NPs	Risperidone	In vivo studies evidenced the prolonged antipsychotic effect of risperidone-loaded biodegradable polymeric nanoparticles compared to risperidone solution administered through intravenous route.	[151]
PLGA	NPs	Risperidone	In vivo studies in mice showed the prolonged antipsychotic effect of risperidone-loaded polymeric nanoparticles up to 72 h with fewer extrapyramidal side effects compared to risperidone solution administered subcutaneously.	[152]
N-iso propylacrylamide, PVP, and acrylic acid in the ratio of 60:20:20	NPs	Curcumin	Polymeric nanoparticles of curcumin displayed higher systemic bioavailability in plasma and tissues compared to free curcumin after parenteral administration. No systemic adverse action was reported.	[153]
Sodium alginate	Nanoparticles	Carboplatin	Prolonged drug release up to 12 h compared to the pure drug (up to 3 h). The drug was detected in the liver, lungs, and spleen after parenteral administration in Laca mice, thereby showing the potential of sodium alginate nanoparticles as a promising tool for targeted drug delivery.	[154]
Methoxypoly (ethylene glycol)-b-poly(*ε*-caprolactone-co-*p*-dioxanone)	Micelles	Curcumin	Polymeric micelles showed slow drug release and dose-dependent inhibition of PC-3 human prostate cancer cells.	[155]
PEG-b-PLA, PEG-b-PCL	Micelles	Sagopilone	Sagopilone-loaded polymeric micelles were found stable up to 24 h at 37 °C. In vitro studies showed high antiproliferative activity (IC_50_ < 1 nM). No carrier-related side effects were observed In vivo.	[156]
PLGA	Nanoparticles	Zaleplon	The developed NPs showed greater ant-convulsant potential in contrast to free drug.	[157]
Poly(propylenesulfide)	Nanoparticles	Diflunisal	The developed NPs resulted in a significant decline in *S. aureus*-mediated bone degradation and aided in establishing the possibility of systematic delivery of anti-viral agents to treat osteomyelitis.	[158]
Chitosan and PCL	Nanoparticles	Paliperidone	The developed formulations showed minimum cellular toxicity.	[159]
Hexadecanol-modified chitosan oligosaccharide	Nanomicelles	Gambogic acid	It was reported that nanomicelles resulted in improved cellular uptake and quick drug release.	[160]
Hyaluronic acid, vitamin E and styrene maleic anhydride	Nanomicelles	Curcumin analogues	The developed nanomicelles revealed excellent TNBC accumulation with minimum spleen and liver retention.	[161]

NPs, nanoparticles; PLGA, Poly(dl-lactide-co-glycolide); PEG, Poly(ethylene glycol); PLA, Polylactic acid.

**Table 5:** A cross-section of reports on polymers employed as nanocarriers for topical delivery of drugs, peptides, and genes.

Polymer	Delivery System	Therapeutic Molecule	Summary	Ref.
Chitosan	NPs	Retinol	Encapsulation of retinol in chitosan nanoparticles improved retinol stability and minimized its irritation and toxicity.	[165]
PCL	NPs	Indomethacin	Ex vivo permeation study of polymeric nanoparticles displayed higher flux across human skin compared to marketed gel formulation, Indotopic^®^ gel.	[166]
PLGA	NPs	Antisense oligodeoxynucleotides	Polymeric nanoparticles displayed 25 to 32% drug release within one day via a diffusion-controlled process followed by PLGA degradation-controlled drug release (39% to 70%) after 14 days.	[167]
Gelatin	NPs	STAT6 siRNA	Gelatin nanoparticles exhibited stable and biocompatible formulation for topical delivery of siRNA.	[168]
Ethyl cellulose, Eudragit^®^ RS	Nanospheres/Nanocapsules	Dexamethasone	In an ex vivo study, polymeric nanoparticles displayed slower drug release and penetration compared to conventional cream and could be an efficient way to control the release and penetration of dexamethasone on the skin and mucous membrane.	[169]
HPMC K15 & PEG 200	NPs	Rosuvastatin	The ex vivo permeation study exhibited significantly higher permeation via rat skin.	[170]

**Table 6:** USFDA-approved polymeric products.

Name	Formulation	Indications	Role	Ref
Polymeric nanoparticles				
Cimzia^®^	PEGylated antibody fragment (Certolizumab)	Crohn’s disease, rheumatoid arthritis, Ankylosing spondylitis	Improved circulation time and greater stability In vivo.	[177]
Krystexxa^®^	Polymer-protein conjugate (PEGylated porcine-like uricase)	Chronic gout	Improved stability of protein through PEGylation	[178]
Plegridy^®^	Polymer-protein conjugate (PEGylated INF)	Multiple sclerosis	Improved protein stability by PEGylation	[179]
Adenovate	Polymer-protein conjugate (PEGylated Factor VIII)	Hemophilia		[180]
Neulasta^®^	PEGylated GCSF protein	Neutropenia		[181]
Pegasys^®^	PEGylated IFN alpha-2a protein	Hepatitis B and C		[182]
Copaxone^®^	Copolymer of L-glutamic acid, L-alanine, L-lysine, and L-tyrosine	Immunomodulator in multiple sclerosis	Improved biocompatibility/solubility	[183]
Polymeric micelles				
Estrasorb™	Micellar estradiol	Menopausal therapy	Controlled delivery	[184]

**Table 7:** A cross-section of reports on polymeric nanocarriers under clinical trials.

Product Developed	Therapeutic Agent	Targeted Disease	Objective	Clinical Trial Status/Verification Date
Polymeric nanoparticle surface modified with somatostatin analog	Cetuximab	Colon cancer and colorectal cancer	To evaluate the bioavailability and therapeutic window.	Phase 1 (Recruiting)/October 2019
Polymeric micelle	Docetaxel	Esophageal Carcinoma	To determine the effects and safety.	Phase 2 (Recruiting)/July 2018
Polymeric micelle	Paclitaxel	Recurrent breast cancer	To examine the response rate.	Phase 4/June 2009
Polymer basednanoparticles	Docetaxel	Advanced solid malignancies	To determine the maximum tolerated dose and evaluate the safety and pharmacokinetics	Phase 1 (completed)/May 2017
Polymeric micelle	Docetaxel	Head and neck squamous cell carcinoma	To determine safety and efficacy	Phase 2 (Recruiting)/April 2017
Polymeric micelle	Paclitaxel	Ovarian cancer	To determine the maximum tolerated dose and evaluate the safety/efficacy	Phase 1 & 2/Dec 2009
Polymeric micelle	Paclitaxel	Non-small cell lung cancer	To examine the response rate.	Phase 2 (completed)/May 2017
Polymeric micelle	Paclitaxel	Bladder cancer and ureter cancer	To examine safety and efficacy	Phase 2 (completed)/December 2011

**Table 8:** A random sample of patent reports on polymeric nanocarriers intended for oral, ocular, topical, and parenteral administration.

Patent Number, Year	Title of the Patent	Description
US 20150353676 A1, 2015	Polymeric nanoparticles and a process of preparation thereof	Disclosed composition and method of preparation for emulsifier-free biodegradable polymeric nanoparticles made of a block copolymer having a size range between 30–120 nm.
US 20150320856 A1, 2015	Method for providing polymeric synthetic nanocarriers for generating antigen-specific tolerance immune responses	Described composition and method of preparation for immunosuppressant loaded pH-sensitive polymeric synthetic nanocarrier, surface engineered with APC antigen for site-specific and controlled drug delivery.
US 20160324966 A1, 2016	Polymeric nanocarriers with a light-triggered release mechanism	Highlighted a method for light-triggered release of PLGA polymeric nanocarrier for biomedical applications.
US 20150079005 A1, 2015	Polymeric nanocarriers with a linear dual response mechanism	Depicted a method for preparation of pH-sensitive polymeric nanocarrier which degrades at lower pH and/or reactive oxygen species.
US 20090258078 A1, 2009	Antioxidant polymer nanocarriers for use in preventing oxidative injury	Presented a composition and preparation method for protein encapsulated polymeric nanocarrier with an intent to protect the protein from protease degradation and increase the therapeutic half-life for topical delivery.
US8613951 B2, 2013	Therapeutic polymeric nanoparticles with mTOR inhibitors and methods of making and using the same	Reveals a preparation method for mTOR inhibitors loaded polymeric nanoparticles made of the diblock copolymer.
US8715741 B2, 2014	Water-dispersible oral parenteral, and topical formulations for poorly water-soluble drugs using smart polymeric nanoparticles	Discloses composition for poorly water-soluble drug encapsulated polymeric nanocarrier conversant with mucoadhesive, oral bioavailability and multifunctional systemic targeting characteristics.

## Data Availability

All the associated data are available within the manuscript.

## Abbreviations

DOX	Doxorubicin
NSM	Nanostructured materials
PCA	polycyanoacrylates
PCL	Polycaprolactone
PDLLA	Poly-DL-lactic acid
PEG	Polyethylene glycol
PGA	poly-glycolic acid
PLA	Polylactic acid
PLGA	Poly-lactic-co-glycolic acid
PVA	Polyvinyl alcohol
QDs	Quantum dots
USFDA	United States Food and Drug Administration.
